# Effect of the m6ARNA gene on the prognosis of thyroid cancer, immune infiltration, and promising immunotherapy

**DOI:** 10.3389/fimmu.2022.995645

**Published:** 2022-11-01

**Authors:** Minqi Xia, Shuo Wang, Yingchun Ye, Yi Tu, Tiantian Huang, Ling Gao

**Affiliations:** ^1^ Department of Endocrinology and Metabolism, Renmin Hospital of Wuhan University, Wuhan, China; ^2^ Department of Breast and Thyroid Surgery, Renmin Hospital of Wuhan University, Wuhan, China

**Keywords:** thyroid cancer, m6A, prognosis, risk model, immune infiltration, immunotherapy

## Abstract

**Background:**

Accumulating evidence suggests that N6-methyladenosine (m6A) RNA methylation plays an important role in tumor proliferation and growth. However, its effect on the clinical prognosis, immune infiltration, and immunotherapy response of thyroid cancer patients has not been investigated in detail.

**Methods:**

Clinical data and RNA expression profiles of thyroid cancer were extracted from the Cancer Genome Atlas-thyroid carcinoma (TCGA-THCA) and preprocessed for consensus clustering. The risk model was constructed based on differentially expressed genes (DEGs) using Least Absolute Shrinkage and Selection Operator (LASSO) and Cox regression analyses. The associations between risk score and clinical traits, immune infiltration, Gene Ontology (GO), Kyoto Encyclopedia of Genes and Genomes (KEGG), Gene Set Enrichment Analysis (GSEA), immune infiltration, and immunotherapy were assessed. Immunohistochemistry was used to substantiate the clinical traits of our samples.

**Results:**

Gene expression analysis showed that 17 genes, except YHTDF2, had significant differences (vs healthy control, *P*<0.001). Consensus clustering yielded 2 clusters according to their clinical features and estimated a poorer prognosis for Cluster 1 (*P*=0.03). The heatmap between the 2 clusters showed differences in T (*P*<0.01), N (*P*<0.001) and stage (*P*<0.01). Based on univariate Cox and LASSO regression, a risk model consisting of three high-risk genes (KIAA1429, RBM15, FTO) was established, and the expression difference between normal and tumor tissues of three genes was confirmed by immunohistochemical results of our clinical tissues. KEGG and GSEA analyses showed that the risk DEGs were related mainly to proteolysis, immune response, and cancer pathways. The levels of immune infiltration in the high- and low-risk groups were different mainly in iDCs (*P*<0.05), NK cells (*P*<0.05), and type-INF-II (*P*<0.001). Immunotherapy analysis yielded 30 drugs associated with the expression of each gene and 20 drugs associated with the risk score.

**Conclusions:**

Our risk model can act as an independent marker for thyroid cancer and provides promising immunotherapy targets for its treatment.

## Introduction

Thyroid cancer is one of the most common tumors in human subjects (1, 2). The main types of thyroid cancer include papillary carcinoma (PTC), follicular thyroid cancer (FTC), undifferentiated thyroid cancer (ATC) and medullary thyroid cancer (MTC) (3). The incidence of thyroid cancer has been increasing (4). At present, the incidence rates of thyroid cancer are 7.4 and 22.0 per 100,000 for males and females in the United States (US), respectively (5). In addition, thyroid cancer is usually asymptomatic (6), and at autopsy, 35.6% of the Finnish population had occult thyroid cancer (7). Currently, most thyroid cancers are curable with conventional treatments such as surgery, radioactive iodide (RAI) therapy,thyroid stimulating hormone (TSH) suppression therapy for local or localized disease (8) and bioinformatics is also a useful tool to analyze sequencing results and clinical data, construct a prognostic model and seek involved pathways or therapeutic targets, particularly in tumor research (9–11). Therefore, using a panel of susceptible genes with RNA/DNA sequencing techniques to identify high-risk patients is advocated for the early diagnosis of high-risk patients and precision medicine (12, 13).

M6A is a ubiquitous RNA modification in eukaryotes (14) and is involved in a variety of biological processes, such as embryonic development, apoptosis, spermatogenesis, and circadian rhythms (15–17). The modification consists of three processes: catalysis, recognition, and removal, which are completed by m6A methyltransferase “writers”, m6A binding protein “readers” and demethylase “erasers”, respectively (18–20). Methyltransferases include METTL3/14, RBM15, RBM15B, WTAP, KIAA1429, and ZC3H13, which are critical in regulating stem cell pluripotency, cell differentiation, and the circadian cycle. RNA-binding proteins, including YTHDC1/2, YTHDF1/2, HNRNPC, ELF3, IGF2BP2 and CBLL1, play an important role in recognizing RNA methylation information and participating in the translation and degradation of downstream RNA. Demethylase consists of FTO and ALKBH5, which mediate RNA demethylation and play a role in energy homeostasis, adipocyte differentiation, and fertility in mice (21–24). Dysregulation of m6A has been linked to cancer progression (25–30). The effect of m6A-related genes on cancers has been widely studied, such as breast cancer (31), gastrointestinal cancer (32), urothelial carcinoma (33), gastric cancer (34). In pancreatic cancer, ALKBH5-mediated upregulation of DDIT4-AS1 maintains pancreatic cancer stemness and inhibits chemosensitivity through activation of the mTOR pathway (35). METTL14 regulates the miR-30c-2-3p/AKT1S1 axis to inhibit gastric cancer progression through mediated mA modifications of circORC5 (36). Less research has been done on m6A in thyroid cancer, but recent studies have revealed that METTL3 inhibits the progression of papillary thyroid carcinoma (37).With the development of recent years, in addition to surgical or conventional treatment, cancer immunotherapy has also made amazing progress in thyroid cancer (38, 39), but the relationship between thyroid cancer and immunity is also unclear.

To further understand the function of m6A-related genes in thyroid cancer, we selected 17 m6A-related genes (40–42) for clinical correlation analysis, immune infiltration analysis and immunotherapy analysis. In this study, thyroid cancer patients were divided into 2 clusters using consensus clustering based on the expression of 17 m6A-related genes. Differences in clinical traits between the two cohorts were analyzed. In addition, we developed a risk model by LASSO regression for predicting overall survival (OS) and further explored the relationship between the risk score and biological processes (BPs), cellular components (CCs), molecular function (MF), pathways, outcomes, immune infiltration and immunotherapy.

## Materials and methods

### Ethics statement

Data for all subjects were obtained from the internet and signed informed consent by default. Analysis of thyroid tissues from this study was carried out under the recommendations of the Regional Ethics Committee guidelines and institutional policies and the Ethics Committee of Renmin Hospital of Wuhan University. Thyroid nodule tissue was obtained from patients with malignant or benign thyroid nodules. All patients signed informed consent forms. Freshly harvested thyroid tissues were used for immunohistochemical staining.

### Data processing

RNA expression quantification data (510 cancers vs. 58 normal) and the corresponding clinical data of the THCA cohort were obtained from the TCGA database (https://portal.gdc.cancer.gov/) (43). The fragments per kilobase of exon model per million mapped fragments (FPKM) values were normalized with the transcripts per million (TPM) method and then converted (log_2_+1) in TCGA. The clinical information of all thyroid patients is shown in **Supplementary Table S1**. The GSE33630 dataset (49 cancers vs. 45 normal) and GSE60542 (33 cancers vs. 35 normal) cohort in our study were downloaded from the GEO database (http://www.ncbi.nlm.nih.gov/geo) (44).The protein-protein interaction (PPI) diagram of 17 m6A-related genes was constructed in the Search Tool for the Retrieval of Interacting Genes/Proteins (STRING) network (https://string-db.org/) (45). PPI genes were analyzed by Cytoscape software. The genetic database used for GSEA data analysis is “MSigdb” (46).

### Differential expression analysis

Differentially expressed genes (DEGs) in normal and tumor thyroid samples were screened by the “limma” package. We extracted the expression matrix of the 17 m6A-related genes, including METTL3/14, RBM15, RBM15B, WTAP, KIAA1429, ZC3H13, YTHDC1/2, YTHDF1/2, HNRNPC, ELF3, IGF2BP2, CBLL1, FTO, and ALKBH5. The R packages “heat-map” and “vioplot” were used to show the differential expression of 17 m6A-related genes in normal and tumor samples. The correlation between 17 m6A-related genes was constructed by the R package “correlation”.

### Protein-protein interaction

We uploaded 17 m6A-related genes to the Search Tool for the Retrieval of Interacting Genes/Proteins (STRING) network to construct a protein-protein interaction (PPI) network to show the association between m6A-related genes.

### The relationship between clusters and clinical traits after clustering analysis

The “ConsensusClusterPlus” package was used to cluster data according to the expression of 17 m6A-related genes in 568 thyroid cancer samples. The consensus matrix and cumulative distribution function (CDF) were used to calculate the optimal number of clusters. The difference in clinical traits between the two clusters was illustrated by the R packages “survival” and “heat-map”.

### Risk model construction and clinical correlation analysis

Univariate Cox analysis was used to evaluate the prognostic value of 17 m6A-related genes with which HR<1 or >1 was regarded as a protective gene or risk gene, respectively. Whether there was an association between genes and prognosis was determined by the *P* value (*p*<0.05). To avoid overfitting, least absolute shrinkage and selection operator (LASSO) regression analysis was performed using the “glmnet” package. The risk score of each patient was calculated by the following formula:


$$\text{Risk Score = }\sum_{i=1}^{n}coef(i)*x(i)$$

Coef(i) and x(i) are regression coefficients and gene expression levels, respectively.

Based on their risk score being below or above the median risk score, 449 THCA samples were divided into two subgroups: the high-risk group and the low-risk group. The distribution of the risk score, survival curve, heatmap of the 3 genes in the model, and receiver operating characteristic (ROC) curve were analyzed. The relationship between the risk score and clinical traits, such as fustat, age, gender, stage, and tumor, nodes and metastases (TNM) stage, was also assessed. A nomogram was created using the package “rms”, and the ROC curve was used to prove the validity of the model. We further combined clinical traits with univariate and multivariate Cox regression analyses using the “survival” package. Analysis variables included age, gender, stage, TNM stage, and risk score. Finally, Cox regression analysis was used to analyze the relationship between each clinical trait and survival probability. The Wilcoxon test was used to calculate the relationship between each clinical trait and risk scores.

### Differential gene enrichment analysis between the high- and low-risk groups

Patients with THCA were divided into high- and low-risk groups based on the median risk score. |log_2_FC|≥1和false discovery rate (FDR)<0.05 as a screening criterion for differential genes between the two groups (high- and low-risk groups). Gene Ontology (GO) and Kyoto Encyclopedia of Genes and Genomes (KEGG) enrichment analyses were performed using the “cluster profile” package based on the selected risk DEGs. GSEA was performed using GSEA version 4.2.3 based on data from the high- and low-risk groups. Set the minimum gene set as 15 and the maximum gene set as 500, and resampling a thousand times, a *P* value of< 0.05 and an FDR of< 0.25 were considered statistically significant.

### Immune infiltration analysis and immunotherapy

We used the R package “limma” of the “normalizeBetweenArrays” function to reduce batch effects that may exist between or within the two cohorts to merge the gene information in GSE33630 and GSE60542.The “ssGSEA” package was used to calculate the immune infiltrating cell score for the TCGA and GEO cohorts, and the score was used to compare immune function and immunological pathways between the high- and low-risk groups, and the relationship between risk score and immune function and immunological pathway was also discussed. To assess the potential impact of the risk score on the immune checkpoint response, we used the “limma” package to calculate the difference between the high- and low-risk groups, |log_2_FC|≥1 and false discovery rate (FDR)<0.05 as significance. We used the data from Genomics of Drug Sensitivity in Cancer (GDSC) (https://www.cancerrxgene.org/) database (47, 48)and The Cancer Therapeutics Response Portal (CTRP)(https://portals.broadinstitute.org/ctrp/) database (49) to obtain the relationship between the expression levels of three genes (RBM15, KIAA1429, FTO) and immunotherapeutic drugs. In addition, drug sensitivity and CCLE expression data were obtained from the PRISM Repurposing dataset (https://depmap.org/repurposing) (50) to assess the relationship between drugs and risk scores.

### Immunohistochemistry

Immunohistochemistry (IHC) was performed as previously described (51). Rabbit antihuman antibodies (FTO antibody (No. DF8421, Affinity Biosciences, Cincinnati, OH, USA), RBM15 antibody (No. DF12061, Affinity Biosciences USA), KIAA1429 antibody (No. 25712-1-AP, Proteintech, Rosemont, IL, USA), were used at 1:500 dilutions. The secondary antibody was peroxidase-labelled antibody (rabbit IgG (H+L) KPL, Baltimore, MD, USA).

## Results

### Research technology road map

**Figure 1:**
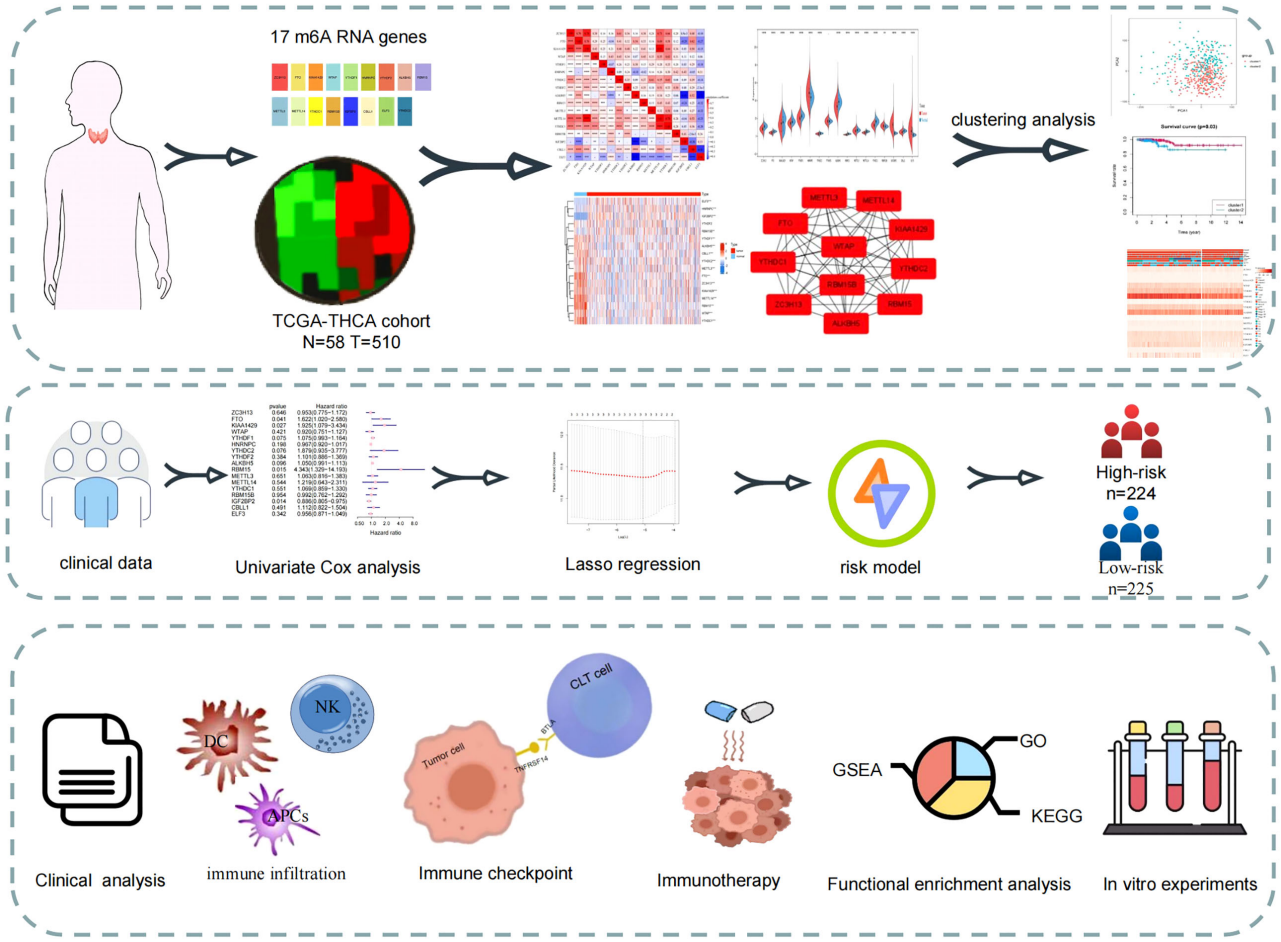
Study flow-chart. Based on the data in TCGA database, the expression data and clinical data were combined to construct a risk model for prognostic analysis, enrichment analysis and immune correlation analysis.

The flow chart of this study is shown in **Figure 1**. By using expression data from the TCGA-THCA cohort, 16 m6A-related genes were selected that differed between normal and tumour samples (|Log_2_FC|>1, FDR<0.05). Risk model based on the three m6A-related genes were then constructed using and LASSO regression analysis, and risk scores were also calculated. Patients were classified into high- and low-risk groups based on the median value of their risk scores in the TCGA-THCA cohort and were further used for subsequent clinical, immune infiltration, mutation, enrichment analysis and immunotherapy analysis. GEO cohort was also used to validation the immune function with risk score. Finally, *in vitro* validation was performed using thyroid cancer and normal tissues.

### Expression of 17 m6A-related genes in the TCGA-THCA cohort

**Figure 2:**
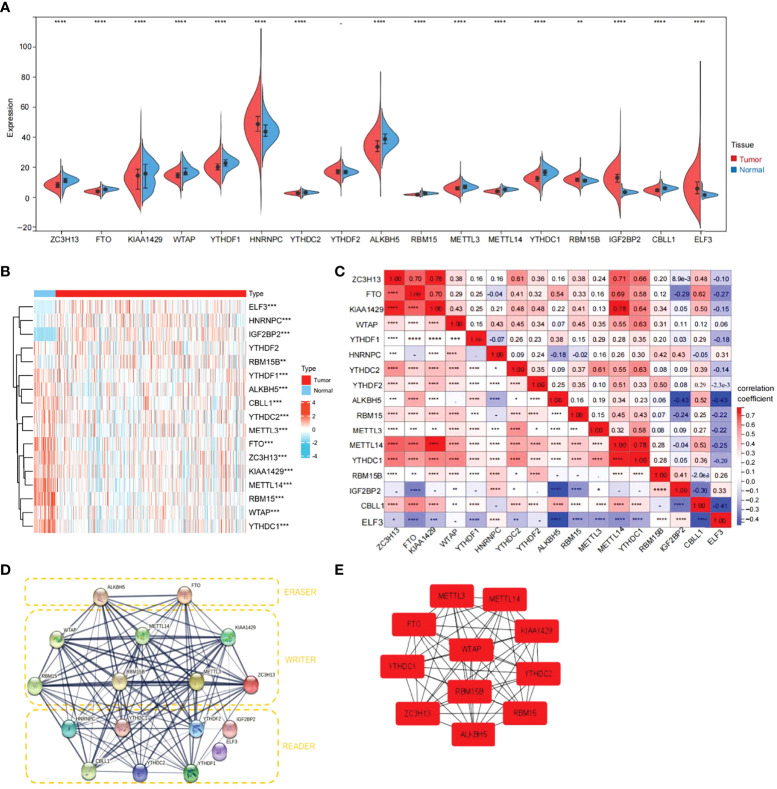
Expression of 17 m6A RNA modification genes in thyroid cancer tissues vs. cancer adjacent tissues and their correlation. **(A)** Vioplot visualizing the 17 m6A RNA methylation genes differentially in thyroid cancer. **(B)** Expression levels of 17 m6A RNA methylation genes (METTL3/14, RBM15,RBM15B, WTAP, KIAA1429, ZC3H13, YTHDC1/2, YTHDF1/2, HNRNPC, ELF3, IGF2BP2, CBLL1, FTO and ALKBH5)in thyroid cancer tissues vs. cancer adjacent tissues (blue bar represents cancer adjacent and red represents thyroid cancer). The higher or lower the expression, the darker the color (red is up-regulated and green is down-regulated). **(C)** The Spearman correlation analysis was used to determine the relationships between each individual 17 m6A RNA methylation genes in thyroid cancer. **(D)** The PPI diagram of 17 m6A RNA methylation genes. **(E)** The cytohubba software analysis yielded the hub genes in the 17 genes. (*p<0.05, **p<0.01, ***p<0.001, ****p<0.0001 was considered significant.)

First, the TCGA-THCA cohort in the TCGA database was analyzed to compare the expression of 17 m6A-related genes in normal and tumor tissues (|Log_2_FC|>1, FDR<0.05). As shown in the heatmap and violin diagram, there were significant differences in 16 genes between normal and tumor tissues, including significantly upregulated expression of HNRNPC, IGFBP2, ELF3, and RBM15B and downregulated expression of ZC3H13, FTO, KIAA1429, WTAP, YTHDC1, YTHDC2, ALKBH5, RBM15, METTL3, METTL14, YTHDC1, and CBLL1 in the TCGA-THCA cohort (**Figures 2A, B**). Spearman correlation analysis further supported the relationship between m6A-related genes (*P*<0.05), such as KIAA1429 and FTO, KIAA1429 and METTL14, ZC3H13 and METTL14, which were significantly positively correlated; ALKBH5 was negatively correlated with IGFBP1; and CBLL1 was negatively correlated with ELF3. Taking KIAA1429 and METTL14 as an example, the correlation coefficient=0.78, *P*<0.0001, indicating that there is a strong correlation between them (**Figure 2C**). A protein-protein interaction (PPI) network of the 17 m6A-related genes was constructed (**Figure 2D**). Next, we used the “Cytohubba” software to calculate the 17 genes and obtained 11 hub genes with the highest scores after calculation: ALKBH5, FTO, METTL14, KIAA1429, RBM15, RBM15B, METTL3, ZC3H13, YTHDC1, YTHDF1, and YTHDC (**Figure 2E**).

### Consensus clustering of 17 m6A-related genes yielded two clusters

**Figure 3:**
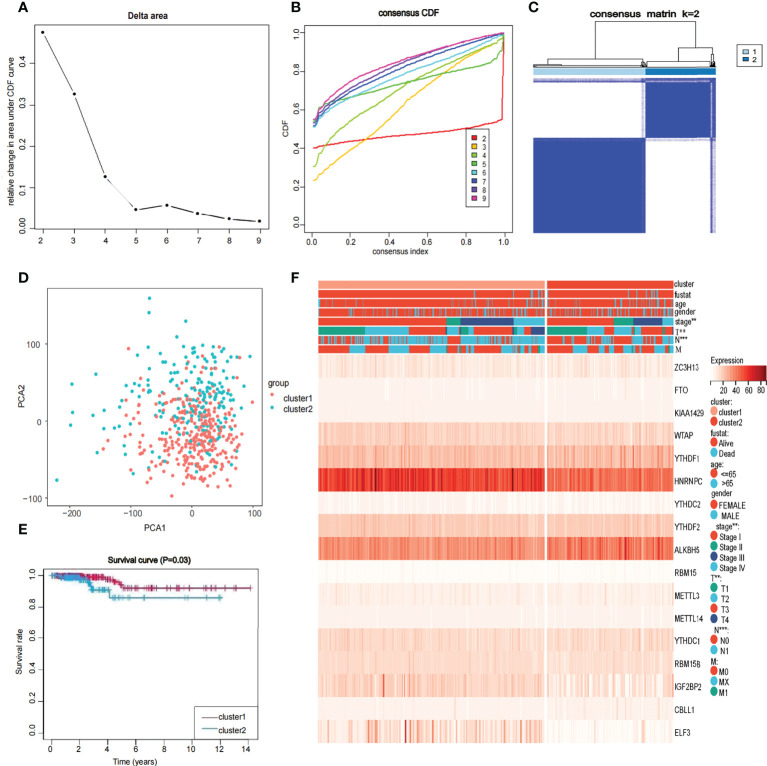
Identification of consensus clusters by 17 m6A RNA modification genes in thyroid cancer **(A, B)** Consensus clustering cumulative distribution function when data is divided into 2-9 clusters. **(C)** Thyroid cancer RNA expression quantification data in TCGA were divided into two different clusters. **(D)** Principal component analysis of the total RNA expression profile in the TCGA dataset. in the cluster 1: red, and the cluster 2: blue. **(E)** The OS of cluster 1 was significantly higher than that of cluster 2 (p<0.05). **(F)** Relationship between 17 genes and clinicopathological factors, including age, gender, stage, fustat, cluster 1 or cluster2, the stage of TNM.

To further understand the overall role of m6A-related genes in the TCGA-THCA cohort, consensus clustering analysis was performed on 568 TCGA-THCA samples based on the expression profiles. Duplicate samples were removed, and similar samples were grouped into one class. To determine the optimal number of clusters, we varied the total number of clusters from 2 to 9 and examined the cumulative density function (CDF) curve of the consensus matrix (**Figures 3A, B**). Finally, K=2 is taken as the optimal cluster number according to the consensus matrix (**Figure 3C**). These subgroups can also be distinguished in principal component analysis (PCA) (**Figure 3D**). The Kaplan-Meier curves showed a significant difference in survival probability between the two clusters, with Cluster 1 having a longer OS (*P*<0.05) (**Figure 3E**). To determine whether there was a significant difference in clinical traits between the two clusters, a clinically relevant heatmap was produced. Based on the heatmap, we observed significant differences in stage (*P*<0.01), T stage (*P*<0.01), and N stage (*P*<0.001) between the two subgroups (**Figure 3F**).

### Construction and value of the risk model

**Figure 4:**
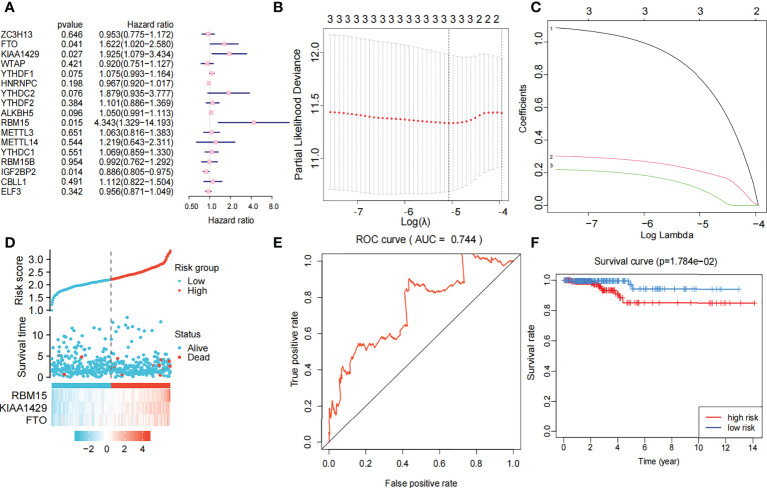
Risk signature with three m6A RNA methylation modulators. **(A)** Univariate Cox regression analysis of the 17 m6A RNA methylation genes in TCGA dataset; the hazard ratios (HR), p value, 3 genes (KIAA1429, FTO, RBM15) are identified (p<0.05, HR>1). **(B, C)** 3 genes (KIAA1429, FTO, RBM15) with p value<0.05 and HR>1 were selected according to the criteria to construct the best predictive gene signatures, and the risk score of the TCGA dataset was calculated using the coefficients obtained by LASSO algorithm. **(D)** Chart of clinical significance-prognostication-risk factors. Compared with the low-risk group, the high-risk group had higher expression levels of 3 genes and higher mortality rate. **(E)** The ROC curve (AUC) of high-risk and low-risk group, the risk signature is credible (AUC=0.744). **(F)** The survival curve of high-risk and low-risk group, the overall survival of patients with thyroid cancer was poor in the high-risk subgroup.

Univariate Cox regression analysis produced four genes (IGFBP2, KIAA1429, RBM15, and FTO). The forest map showed that KIAA1429, RBM15, and FTO were risk factors (HR>1), and IGFBP2 was a protective factor (HR<1). IGFBP2 was not included in the LASSO regression analysis because it was not a hub gene in 17 m6A-related genes (**Figure 4A**). Three risk factors (KIAA1429, RBM15, and FTO) were selected, and LASSO regression analysis was performed. These 3 genes (KIAA1429, RBM15, and FTO) were retained according to the minimum partial likelihood bias (**Figures 4B, C**). For the TCGA-THCA cohort, the risk score was calculated by the following formula:


Risk Score = (0.6518∗RBM15 expression) + (0.1832∗ KIAA1429 expression) + (0.0507 ∗  FTO expression)

**Figure 5:**
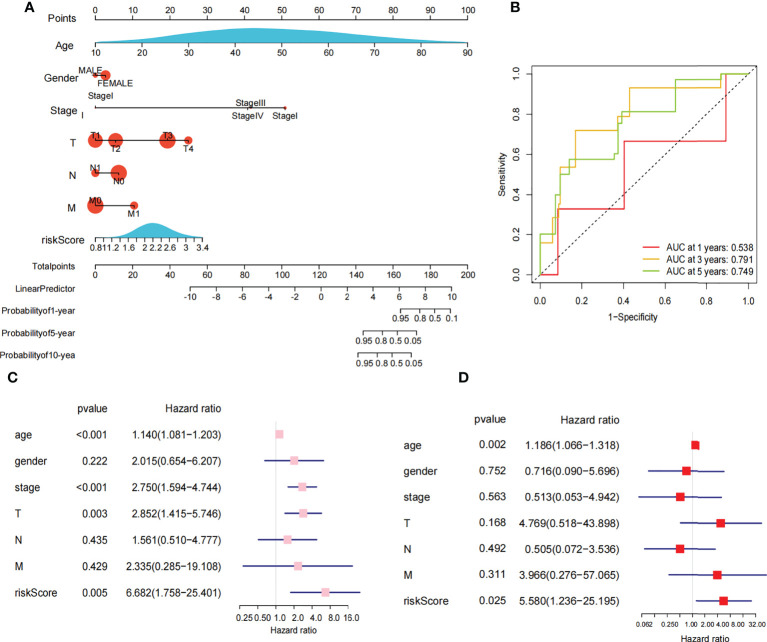
Nomogram and cox regression analysis. **(A)** Novel prognostic nomogram was built by using the risk score and clinical features, such as gender, risk, stage and age. **(B)** The ROC curve analysis of 1 year,5years and 10 years. **(C)** Univariate Cox regression analysis of the association between clinicopathological factors (including the risk score) and overall survival of patients in the group of high risk and low risk. **(D)** Multivariate Cox regression analysis of the association between clinicopathological factors (including the risk score) and overall survival of patients in the in the group of high risk and low risk.

The 449 samples were scored according to a risk scoring formula and were classified as high-risk and low-risk by the median of the risk score (there were 224 high-risk samples and 225 low-risk samples, with the middle 2 values 2.209 and 2.211, respectively). We also described the sample distribution for the three m6A-related genes, risk assessment subgroups, and survival conditions (**Figure 4D**). The accuracy of the risk score was evaluated by calculating the area under the ROC curve (AUC=0.744) (**Figure 4E**). There were significant differences in survival probability between the high- and low-risk groups (*P*=1.784e-02), and the overall survival of patients with thyroid cancer was poor in the high-risk group (**Figure 4F**). We constructed a prognostic nomogram using risk score and clinical traits such as survival, age, gender, stage, T stage, N stage and M stage. (**Figure 5A**). The c-index of the model population was 0.959 95% confidence interval (CI) (0.939-0.987), *P* value=1.091e-223. The nomogram can effectively predict the overall survival time of patients with THCA. ROC curve analysis showed that the AUC was 0.538 at 1 year, 0.791 at 5 years, and 0.749 at 10 years (**Figure 5B**). Then, univariate Cox regression analysis showed that age, stage, T stage and risk score were correlated with the prognosis of patients (*P*<0.05). Multivariate Cox regression analysis showed that age and risk score could be independent prognostic indicators (*P*<0.05) (**Figures 5C, D**).

### Clinical subgroup analysis

**Figure 6:**
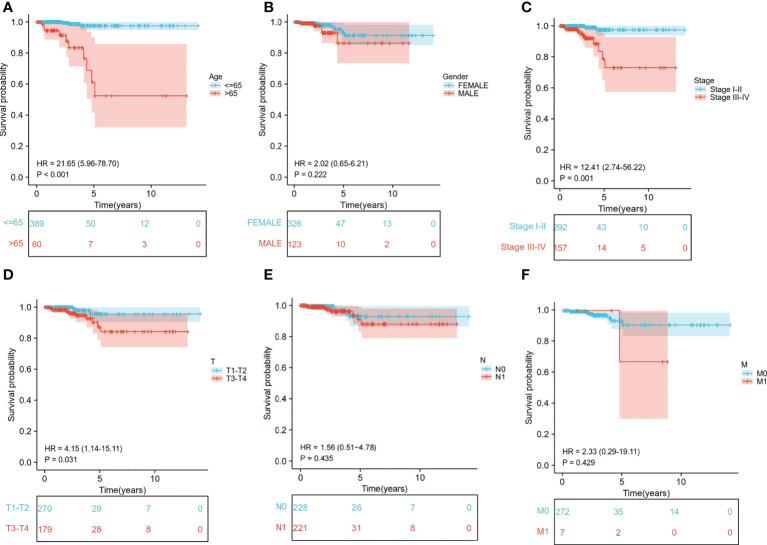
Relationship between clinical subgroups and survival probability. **(A)** Difference in survival probability between age > 65 years and age ≤65 years. **(B)** Difference in survival probability between Male and Female. **(C)** Difference in survival probability between Stage I-II and Stage III-IV. **(D)** Difference in survival probability between T1-T2 and T3-T4. **(E)** A. Difference in survival probability between N0 and N1. **(F)** Difference in survival probability between M0 and M1.

We extracted the clinical data of age, gender, stage, and TNM stage to further explore the relationship between clinical traits and survival probability. Then, we eliminated the samples for which information was missing and carried out Cox regression analysis on the remaining samples to obtain the clinical traits and survival probability as well as the relationship between the clinical traits and risk score. According to our findings, age>65 had a significantly lower chance of survival than age ≤ 65 (*P*<0.001) (**Figure 6A**). Stage I-II, T1-T2 and M0 patients had a significantly higher chance of surviving than stage III-IV, T3-T4 and M1 patients (*P<*0.05). In contrast, neither gender nor N stage seemed to significantly affect survival probability (**Figures 6B-F**). Regarding the differences in clinical traits between the high- and low-risk groups, the high- and low-risk groups’ survival probabilities were significantly different (*P*<0.05) for patients who were over 65 and in Stages III-IV (**Supplementary Table S2**). Additionally, the risk scores between the T1-T2 and T3-T4 groups differed significantly (*P*<0.05). However, other clinical traits (age, gender, stage, N, and M) did not appear to be associated with a difference in risk scores (**Supplementary Table S3**).

### Functional analysis based on the risk model

**Figure 7:**
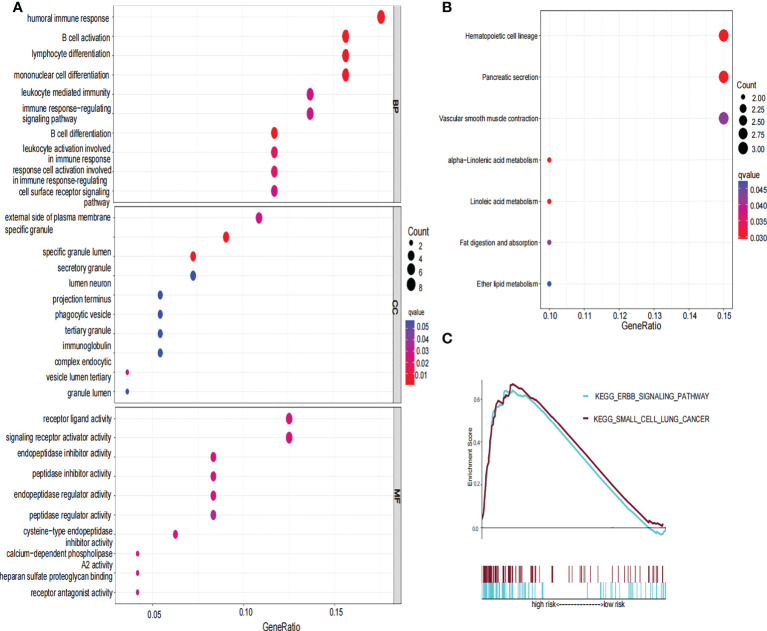
GO, KEGG and GSEA analysis of risk signature. **(A)** The GO diagram was made according to the high- and low-risk group. **(B)** The KEGG diagram was made according to the high and low-risk group. **(C)** Gene set enrichment analysis was performed according to the high and low-risk group.

To further explore the functional difference between the high- and low-risk groups, we used the R package “limma” to screen DEGs, and the screening standard was set to FDR<0.05 and |log_2_FC|≥1. A total of 76 DEGs were screened between the high-risk and low-risk groups in the TCGA cohort. Among these genes, 49 genes in risk DEGs were upregulated, and the remaining 27 genes were downregulated. Next, we performed GO, KEGG, and GSEA analyses based on 76 risk DEGs. GO and KEGG analyses showed that risk DEGs were related mainly to proteolysis, lipid metabolism, and immune response (**Figures 7A, B**). GSEA showed that risk DEGs were related mainly to the cancer pathway (**Figure 7C**).

### Immune infiltration and immunotherapy

**Figure 8:**
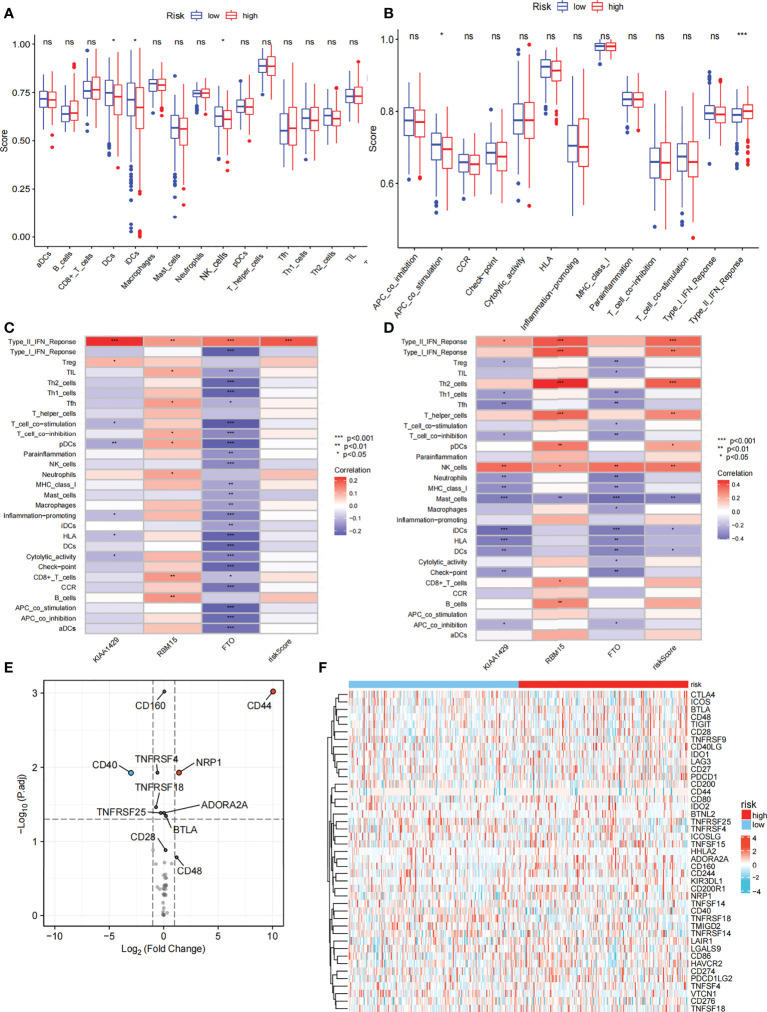
Immune function and immune checkpoint analysis. **(A)** Correlation between high and low-risk group and various immune cells in thyroid cancer and adjacent tissues. **(B)** Correlation between high and low-risk group and various immune functions in thyroid cancer and adjacent tissues. **(C)** The relationship between risk score and immune cells and functions in TCGA cohort. **(D)** The relationship between risk score and immune cells and functions in GEO cohort. **(E)** Volcano plot of the distribution of immune checkpoint-associated genes in high- and low-risk groups. **(F)** Expression of the immune checkpoint in the high-and low-risk groups. ("ns" means “no significance” *p<0.05, **p<0.01, ***p<0.001, p<0.05 was considered significant).

**Figure 9:**
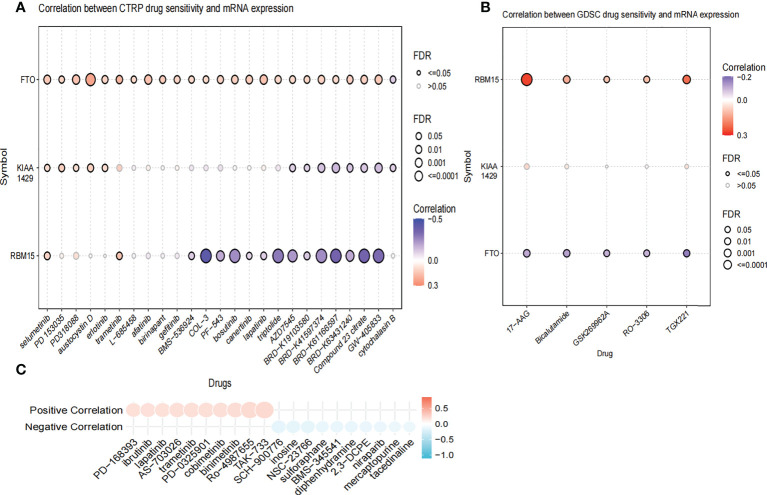
The Relationship between risk scores and drug sensitivity. **(A)** The relationship between risk score and drug sensitivity in the CTRP database. **(B)** The relationship between risk score and drug sensitivity in the GDSC database. **(C)** The relationship between risk score and drug sensitivity in the PRISM database.

We further compared the enrichment fractions of immune cells and the activity of immune-related pathways between the high- and low-risk groups in the TCGA-THCA cohort. A comparison of the various infiltrative immune cells in tumors showed that the degree of DC, iDC and NK-cell infiltration was higher in the low-risk group than in the high-risk group, while other immune cells did not differ between the two groups (**Figure 8A**). In the TCGA cohort, the activity of the Type_II_IFN pathway was significantly higher than the activity of the low-risk group, while the activity of the APC_costimulation pathway was higher in the low-risk group. The other 11 immune pathways showed little difference between the high-risk and low-risk groups (**Figure 8B**). Next, we further investigated the relationship between risk scores and individual immune cells and validated it using GEO data. TCGA data showed that risk score was significantly positively correlated with Type-II-IFN-Response pathway, and this conclusion was also confirmed in GEO database (**Figures 8C, D**). We used volcano plots to show the differences in immune checkpoints between high- and low-risk groups, P value of < 0.05 and |log_2_FC|>1 were considered statistically significant (**Figure 8E**). Furthermore, we mapped the relationship between the risk groups and immune checkpoints (**Figure 8F**). Using the GDSC and CTRP databases, we summarized the correlation between gene expression and drug sensitivity across cancers, and the 30 drugs with the strongest correlation are listed (**Figures 9A, B**). Furthermore, we analyzed the association between risk scores and responsiveness to nontumor drugs through the PRISM database (**Figure 9C**). The results showed that 20 drugs, including PD-168393, ibrutinib, lapatinib, AS703026, trametinib, PD-0325901, cobimetinib, binimetinib, RO4987655, TAK733, SCH900776, inosine, NSC23766, sulforaphane, BMS3445541, diphenhydramine, 2,3-DCPE, niraparib, mercaptopurine and tacedinaline, had correlations with risk scores.

### Expression of RBM15, KIAA1429, and FTO in benign thyroid nodule tissue vs thyroid cancer tissues

**Figure 10:**
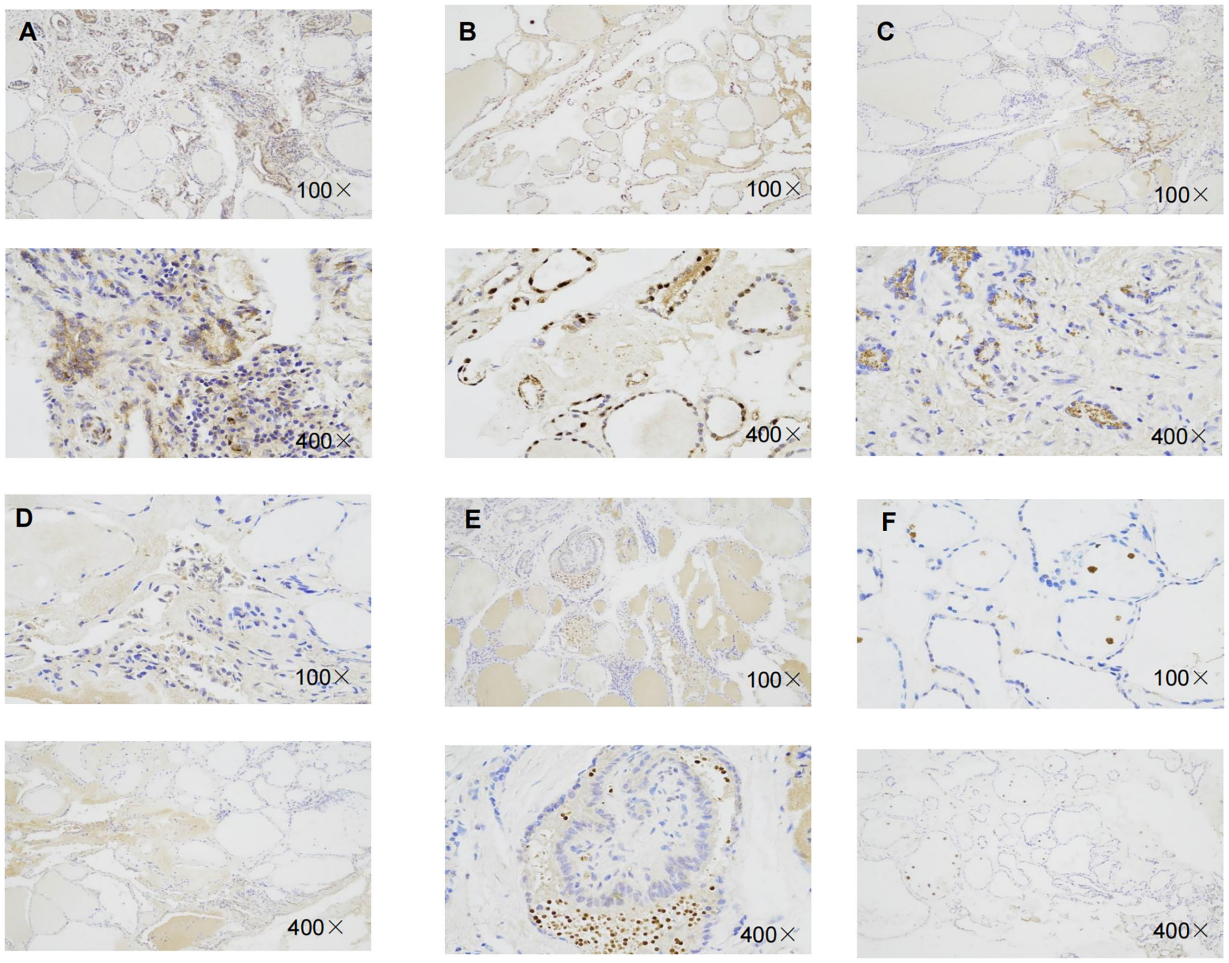
Representative immunohistochemical staining of RBM15, KIAA1429, FTO in benign thyroid nodule tissue and thyroid cancer tissue. **(A)** Immunohistochemical image of FTO in thyroid cancer tissue. **(B)** Immunohistochemical image of FTO in benign thyroid nodule tissue.**(C)** Immunohistochemical image of RBM15 in PTC tissue. **(D)** Immunohistochemical staining image of RBM15 in benign thyroid nodule tissue. **(E)** Immunohistochemical image of KIAA1429 in PTC tissue. **(F)** Immunohistochemical image of KIAA1429 in benign thyroid nodule tissue.

Immunohistochemistry result revealed that the staining intensity of 3 genes (RBM15, FTO, and KIAA1429) were higher in thyroid cancer tissue than in benign thyroid nodule tissue in our specimens which is consistent with bioinformatic analysis (**Figure 10**).

## Discussion

In this study, based on data from the TCGA-THCA cohort, we obtained DEGs between normal and tumor tissues and identified 3 m6A-related genes (KIAA1429, RBM15, and FTO) to construct a risk model and verified its credibility. Based on the risk model, the differences in enrichment pathways, immune function, and immunotherapy between the high- and low-risk groups were compared. Small molecule drugs can be designed for intervention based on the risk score and immune checkpoints, which has the potential to improve the treatment of chemotherapy, radiotherapy, and even immunotherapy. The risk model also provides a new possible avenue for exploring the treatment of thyroid cancer based on the risk score.

We analyzed the expression of 17 m6A-related genes in thyroid cancer and adjacent tissues and found that except for YTHDF2, the other 16 genes were differentially expressed in thyroid cancer. In particular, HNRNPC, RBM15B, IGFBP2, and ELF3 were upregulated, while the other 12 genes were downregulated in thyroid cancer tissues. In addition, there were multiple correlations between m6A-related genes. For example, KIAA1429 and FTO, KIAA1429 and METTL14, and ZC3H13 and METTL14 were significantly positively correlated. ALKBH5 was negatively correlated with IGFBP1 and CBLL1 with ELF3. The correlation between some of these genes has been reported, which is consistent with the conclusions of this paper (52–55).

Based on the expression of 17 m6A-related genes in TCGA-THCA cohort, we identified two distinct molecular clusters. Compared with Cluster 1, Cluster 2 had worse survival probability. The results showed that there were significant differences between the two groups in the clustering analysis. Therefore, we further built the risk model. To further select genes related to prognosis and establish a risk model, we used univariate Cox analysis to select 3 genes with unfavorable prognosis: RBM15, KIAA1429, and FTO. LASSO regression analysis was further used to calculate the risk scores of each clinical dataset, and the data were divided into low- and high-risk groups based on the median risk scores of all samples. Three genes for constructing the risk model have been shown to be associated with cancer. A study on breast cancer showed that KIAA1429 can be a carcinogenic factor of breast cancer by regulating CDK1 independently of m6A (56). RBM15 is an important factor in X chromosome silencing and is expressed in breast tissue (40, 57–59). Studies have also shown that estrogen can independently increase the expression of the RBM15 gene and is associated with the occurrence of breast diseases (59). The FTO gene is highly expressed in breast cancer (60), acute myeloid leukaemia (AML) (27), and glioblastoma (61) and promotes the progression of these cancers, and related drugs have been developed. However, FTO showed low expression in bladder cancer (62). The carcinogenic role of FTO seems to be controversial. In addition to being associated with cancer, FTO is also associated with immunity. KIAA1429 has been shown to affect a variety of cancers through different pathways, including promoting the progression of hepatocellular cancer through the posttranscriptional modification of m6A relying on GATA3 and regulating cell proliferation in gastric cancer by directly targeting c-jun mRNA (62, 63). Immunosuppressants have been reported to be widely used in the clinical treatment of malignant tumors, including thyroid cancer (64). RBM15 expression was positively correlated with immune-infiltrating cells in renal clear cell carcinoma (KIRC), brain low-grade glioma (LGG), and pancreatic adenocarcinoma (PAAD). In addition, RBM15 expression was strongly correlated with immune checkpoint markers in PAAD (65). In addition, some studies have found that downregulation of FTO in the chorionic villi destroys immune tolerance and angiogenesis at the maternal-fetal interface, leading to abnormal methylation and oxidative stress and ultimately to spontaneous abortion (66).

We found that the survival rate of the high-risk group was significantly lower than that of the low-risk group. Next, we constructed a nomogram to further improve the predictive power, which showed that the predictive nomogram could be applied to the TCGA-THCA cohort. As the total clinical score increased, the survival probability of the patient gradually decreased. The C-index of this nomogram reached 0.959, indicating the reliability of the nomogram. We also analyzed the differences in survival probability for each clinical subgroup, and there were significant differences in survival among age, stage, and T-stage subgroups. In order to explore the impact of multiple clinical traits and risk score on the prognosis of patients, we further performed a multivariate Cox analysis, which showed that age and risk score were indeed independent risk factors for the prognosis of thyroid cancer patients. The risk score, as an independent risk factor for prognosis, has also been studied in other diseases (40).In conclusion, this risk model has higher diagnostic power than previous cluster analyses and is more helpful in assessing patient prognosis.

In addition, according to the GO, KEGG and GSEA analyses of the DEGs in the high- and low-risk groups, the analysis results showed that the functions of the risk DEGs were related mainly to proteolysis, immune response, and cancer pathway. In the process of tumor migration, proteolytic enzymes can degrade the basement membrane, make cancer cells through the basement membrane, and migrate elsewhere, in which proteolytic enzymes play an important role in the process of tumor invasion and metastasis. For example, some proteases in thyroid cancer are elevated in tumors (67):transmembrane protease serine 4 promotes thyroid cancer proliferation through cAMP response element-binding protein (CREB) phosphorylation (68), and the HIV protease inhibitor nefinavir induces apoptosis of thyroid medulla cancer cells by downregulating RET signalling (69). These results suggest that the formation mechanism of thyroid cancer in the high-risk group was related to the action of proteolytic enzymes. In our study, the GO results showed that differences in immune function between the high-risk and low-risk groups were manifested mainly in monocyte differentiation, lymphocyte differentiation and B-cell activation. GSEA showed that there were significant differences between the high-risk and low-risk groups in the ERBB pathway and small cell lung cancer pathway, which overlapped with our results of immune function analysis and further demonstrated that our prognostic model was inseparable from cancer and immunity.

Currently, the treatment of thyroid cancer is limited mainly to surgical resection (70, 71), which can improve the survival probability of patients. However, the treatment of thyroid cancer has expanded, and great progress has been made with anticancer drugs. Immunotherapy has become an important part of thyroid cancer treatment (72–77). Therefore, we performed immune analysis for the high- and low-risk groups. In this study, compared with the low-risk group, the immune infiltration levels of dendritic cells, iDCs and NK cells in the high-risk group were lower, but Type II IFN pathway activity was higher. Moreover, we can see that Type II IFN pathway activity are significantly positively correlated with risk score in both TCGA and GEO cohorts. The decrease in dendritic cells, iDCs and NK cells, which are key cells in the immune response and suppress the development of primary tumors and metastases (78–82), may be related to the lower survival rate in the high-risk group. Type II IFN has a rejection effect on highly immunogenic tumors (83). IFN-γ, the only type II IFN, binds to the type II IFN receptor and activates Jak1 and Jak2, which then further activates STAT1. The activated STAT1 homodimer binds to the IFN-γ activation site in the promoter of some ISGs to regulate immune function (84). However, it has also been shown that long-term exposure to IFN-γ can lead to immune escape due to cell desensitization and immune editing (85, 86). Its expression was elevated in the high-risk group, suggesting that Type-II IFN is involved in the antithyroid cancer effect in the high-risk group of thyroid cancer. The increased expression of Type-II IFN in high-risk group indicates that it may be involved in the anti-thyroid cancer effect, but it cannot exclude that it is involved in the immune escape of thyroid cancer.

Compared with the low-risk group, CD44 and NPR1 were significantly increased in the high-risk group. CD44 is encoded by 20 exons that are alternatively spliced to generate the CD44 standards (CD44s) and the CD44 variants (CD44v) (87). Recent studies have shown that abnormal levels and variant forms of CD44 are expressed in a variety of tumor types (88–95). High expression of CD44 is thought to be associated with poor prognosis in cancer, and in addition to this, CD44 v6 and v3 have been reported to be associated with cancer metastasis. Inhibition of CD44 has a cancer-inhibiting effect. It has been shown that inhibition of CD44 inhibits the development of colon tumors (96) in mice and suppresses the proliferation and metastasis of liver and ovarian cancer cells (97, 98). NRP1 is also involved in the cell proliferation, migration, and invasion associated with cancer progression (99). In colorectal cancer, inhibition of NRP1 was able to inhibit metastasis of colorectal cancer cells (100). Moreover, NRP1/mdm2-targeted d-peptide supramolecular nanodrugs have been shown to be highly effective and less toxic for the treatment of hepatocellular carcinoma, with strong anti-cancer activity against SK-Hep-1 cells *in vitro* and *in vivo*, without significant host toxicity, making them a promising treatment for hepatocellular carcinoma (101). In summary, the CD44 and NRP1 inhibitors to treat cancer patients with high-risk scores are promising. From the perspective of the relationship between immunotherapy drugs and risk score, the drugs for patients with high risk score mainly focus on the class of teninib drugs, and these drugs may provide new insights into the treatment of thyroid cancer.

Finally, we verified the expression of 3 genes in thyroid cancer and thyroid nodule tissues by immunohistochemistry. Compared with the thyroid nodule tissues, the expression of 3 genes was increased in thyroid cancer tissues, indicating that the 3 genes constructed as models were also associated with the development of thyroid cancer.

## Conclusion

Based on bioinformatics and *in vitro* experiments, we determined that there were differences in the expression of the m6A-related gene in thyroid cancer. The risk model was able to predict the survival probability of patients with thyroid cancer and confirmed that the risk score was an independent risk factor for thyroid cancer. In addition, enrichment analysis of the high-low risk group confirmed that there were significant differences in proteolysis, immune response, and cancer formation in the high/low-risk group. Further analysis of immune function showed that there were differences in DCs, iDCs, NK cells, and Type II IFN response between the two groups. In addition, immune checkpoint analysis yielded two immune (CD44 and NRP1) checkpoints with therapeutic potential. Finally, the immunodrug analysis identified 20 drugs that were associated with risk scores.

There is no doubt that there are some limitations to the study. First, all analyses were based on retrospective data from a public database. In addition, we have only performed a simple study on the relationship between BP, CC, MF, pathway, outcome, immune function and immunological pathways, immune checkpoint, and THCA and the actual effects of proteolytic, iDCs, NK cells, and Type-II IFN. However, this study systematically analyzed the expression differences of m6A-related genes in thyroid cancer and constructed a risk model that could evaluate the survival, prognosis, and immunotherapy of patients.

## Data availability statement

The raw data supporting the conclusions of this article will be made available by the authors, without undue reservation.

## Ethics statement

The studies involving human participants were reviewed and approved by Ethics Committee of Renmin Hospital of Wuhan University. The patients/participants provided their written informed consent to participate in this study.

## Author contributions

MX and TH performed the data analysis and wrote the manuscript. SW ,YY,LG participated in the study design, figures,writing and revised the manuscript. YT provided tissue samples.All authors read and approved the final manuscript.

## Funding

LG supported from the National Natural Science Foundation of China (No. 81571376), the Fundamental Research Funds for the Central Universities, #2042020kf1079 and China Young Scientific Talent Research Fund for diabetes 2017.

## Conflict of interest

The authors declare that the research was conducted in the absence of any commercial or financial relationships that could be construed as a potential conflict of interest.

## Publisher’s note

All claims expressed in this article are solely those of the authors and do not necessarily represent those of their affiliated organizations, or those of the publisher, the editors and the reviewers. Any product that may be evaluated in this article, or claim that may be made by its manufacturer, is not guaranteed or endorsed by the publisher.

## Supplementary material

Supplementary Table 1 The baseline information of patients. The clinical information of all thyroid patients, including survival status, age, gender, stage, T stage,N stage and M stage.

Supplementary Table 2 Differences in survival probability between high - and low-risk groups in each clinical subgroup. **A.** Differences in survival between high - and low-risk groups at age ≤65 years. **B.** Differences in survival between high - and low-risk groups in female. **C.** Differences in survival between high - and low-risk groups in Stage I-II.**D.** Differences in survival between high - and low-risk groups at age > 65 years. **E.** Differences in survival between high - and low-risk groups in male. **F.** Differences in survival between high - and low-risk groups in Stage I-II.**G.** Differences in survival between high - and low-risk groups in T1-T2 stage. **H.** Differences in survival between high - and low-risk groups in N0 stage. **I.** Differences in survival between high - and low-risk groups in M0 stage. **J.** Differences in survival between high- and low-risk groups in T3-T4 stage. **K.** Differences in survival between high - and low-risk groups in N1 stage. **L.** Differences in survival between high - and low-risk groups in M1 stage.

Supplementary Table 3 The differences in risk scores between clinical subgroups **A-F.** Differences in risk scores among clinical characteristics subgroups (age, sex, stage, TNM stage). (* p<0.05, ** p<0.01, *** p<0.001, p<0.05 was considered significant.)
