# Supplementary material for: Effect of the m6ARNA gene on the prognosis of thyroid cancer, immune infiltration, and promising immunotherapy
Source: Front Immunol. 2022 Nov 1;13:995645. doi: 10.3389/fimmu.2022.995645 (PMC9664221; doi:10.3389/fimmu.2022.995645)
Supplement: Supplementary Table 1 — The baseline information of patients. The clinical information of all thyroid patients, including survival status, age, gender, stage, T stage,N stage and M stage. [file Table_1.pdf]

| Clinical Characteristic | TCGA-THCA (n=455) | Percentage |
|---|---|---|
| **Survival status** | | |
| Alive | 436 | 97.10% |
| Dead | 13 | 2.90% |
| **Age** | | |
| <=65 | 389 | 86.64% |
| >65 | 60 | 13.36% |
| **Gender** | | |
| FEMALE | 326 | 72.61% |
| MALE | 123 | 27.39% |
| **Stage** | | |
| Stage I | 249 | 55.46% |
| Stage II | 43 | 9.58% |
| Stage III | 104 | 23.16% |
| Stage IV | 53 | 11.80% |
| **T** | | |
| T1 | 130 | 28.95% |
| T2 | 140 | 31.18% |
| T3 | 157 | 34.97% |
| T4 | 22 | 4.90% |
| **N** | | |
| N0 | 228 | 50.78% |
| N1 | 221 | 49.22% |
| **M** | | |
| M0 | 272 | 97.49% |
| M1 | 7 | 2.51% |
