# Supplementary figures and images for: Effect of the m6ARNA gene on the prognosis of thyroid cancer, immune infiltration, and promising immunotherapy
Source: Front Immunol. 2022 Nov 1;13:995645. doi: 10.3389/fimmu.2022.995645 (PMC9664221; doi:10.3389/fimmu.2022.995645)

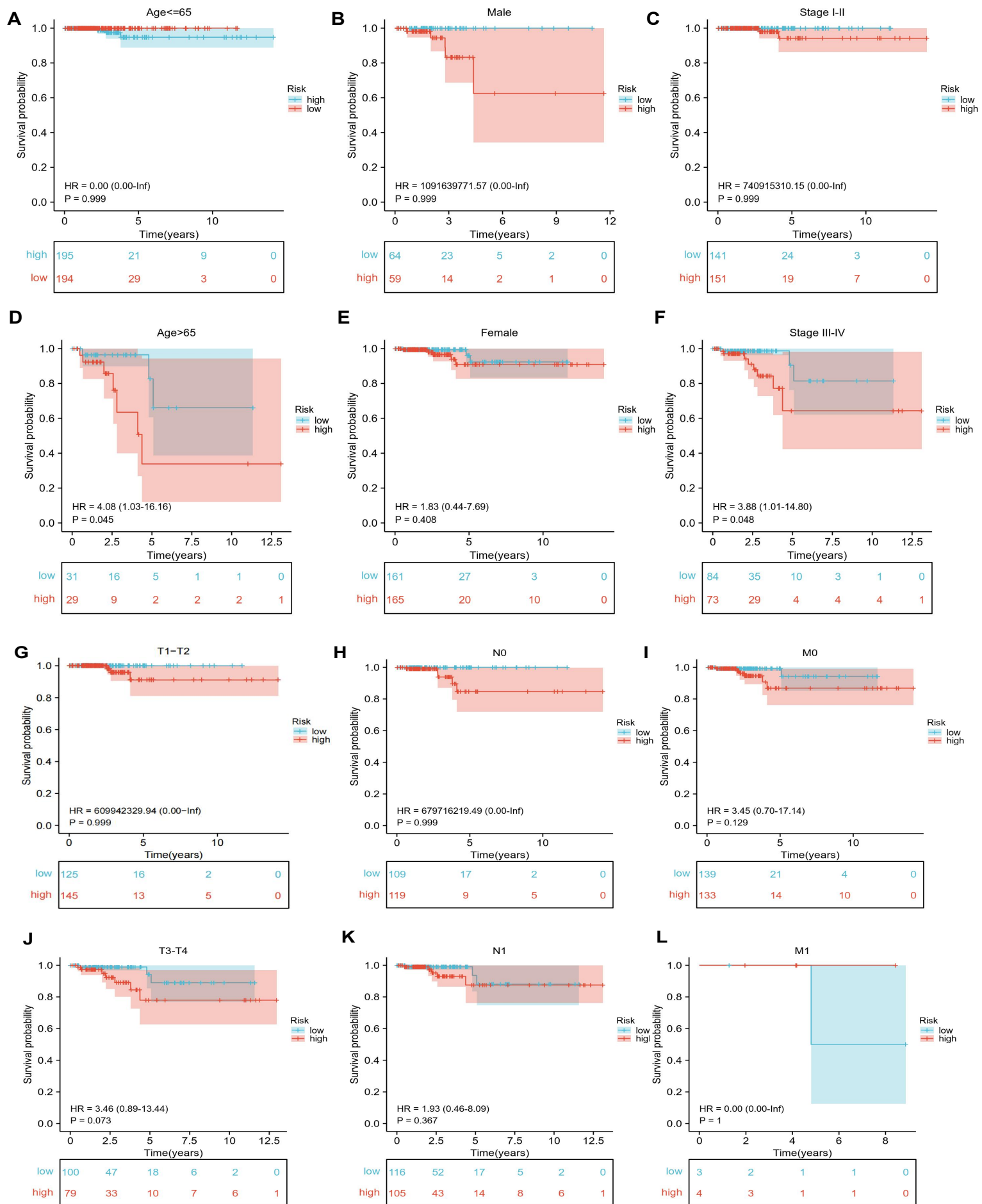

Supplement: Supplementary Table 2 — Differences in survival probability between high - and low-risk groups in each clinical subgroup. A. Differences in survival between high - and low-risk groups at age ≤65 years. B. Differences in survival between high - and low-risk groups in female. C. Differences in survival between high - and low-risk groups in Stage I-II.D. Differences in survival between high - and low-risk groups at age > 65 years. E. Differences in survival between high - and low-risk groups in male. F. Differences in survival between high - and low-risk groups in Stage I-II.G. Differences in survival between high - and low-risk groups in T1-T2 stage. H. Differences in survival between high - and low-risk groups in N0 stage. I. Differences in survival between high - and low-risk groups in M0 stage. J. Differences in survival between high- and low-risk groups in T3-T4 stage. K. Differences in survival between high - and low-risk groups in N1 stage. L. Differences in survival between high - and low-risk groups in M1 stage. [file Table_2.pdf]

**A**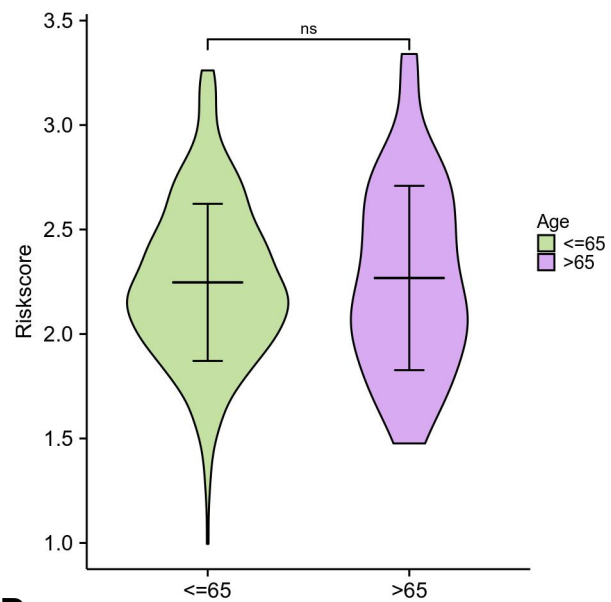**B**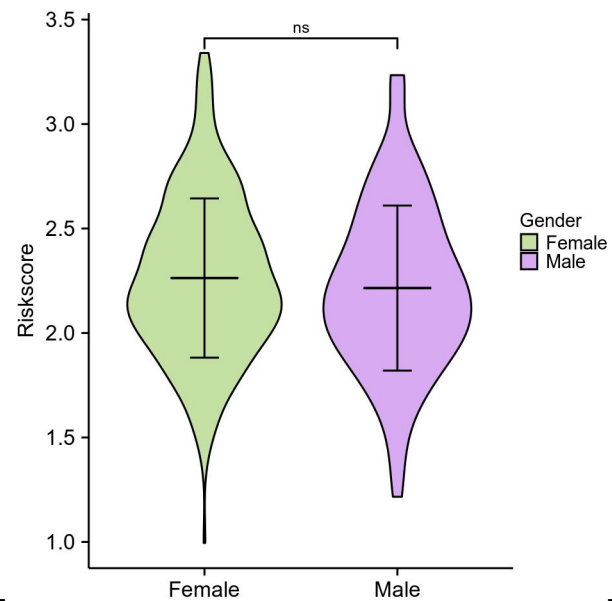**C**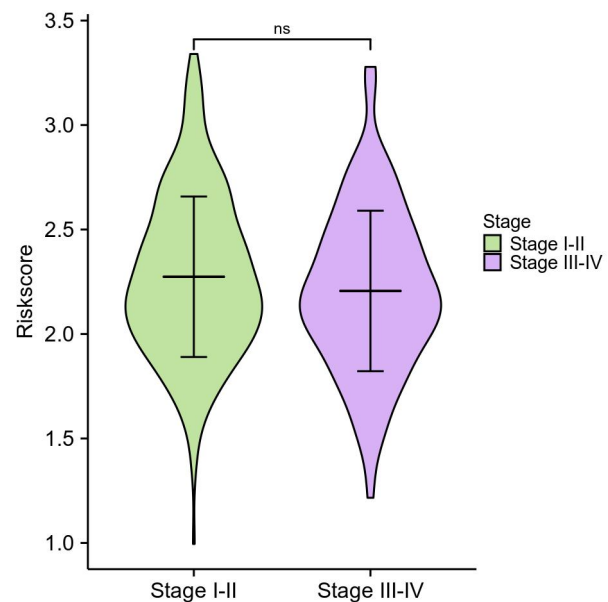**D**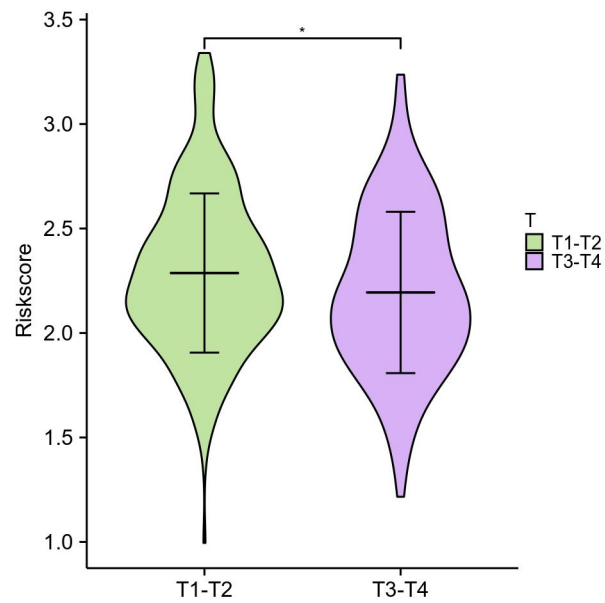**E**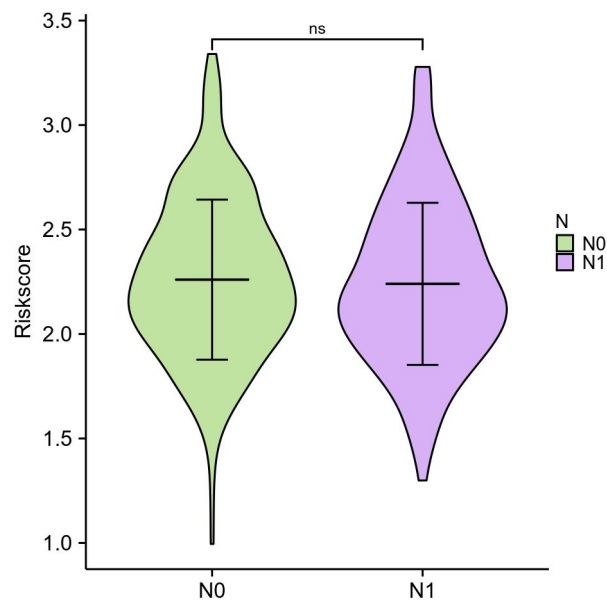**F**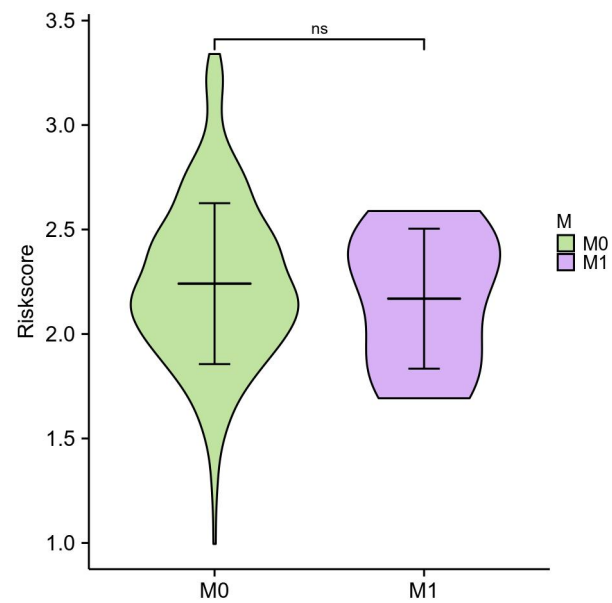

Supplement: Supplementary Table 3 — The differences in risk scores between clinical subgroups A-F. Differences in risk scores among clinical characteristics subgroups (age, sex, stage, TNM stage). (* p<0.05, ** p<0.01, *** p<0.001, p<0.05 was considered significant.) [file Table_3.pdf]
